# Re-sequencing of the APOAI promoter region and the genetic association of the -75G > A polymorphism with increased cholesterol and low density lipoprotein levels among a sample of the Kuwaiti population

**DOI:** 10.1186/1471-2350-14-90

**Published:** 2013-09-12

**Authors:** Suzanne A Al-Bustan, Ahmad E Al-Serri, Babitha G Annice, Majed A Alnaqeeb, Ghada A Ebrahim

**Affiliations:** 1 Department of Biological Sciences, Faculty of Science, Kuwait University, PO Box 5069, Safat 13060, Kuwait; 2 Department of Molecular Pathology, Faculty of Medicine, Kuwait University, Safat Kuwait; 3 Yarmouk Clinic, Ministry of Health, Safat Kuwait

**Keywords:** APOAI, Polymorphism, LDL, Re-sequencing, SNPs, Arabs, Kuwait

## Abstract

**Background:**

APOAI, a member of the APOAI/CIII/IV/V gene cluster on chromosome 11q23-24, encodes a major protein component of HDL that has been associated with serum lipid levels. The aim of this study was to determine the genetic association of polymorphisms in the APOAI promoter region with plasma lipid levels in a cohort of healthy Kuwaiti volunteers.

**Methods:**

A 435 bp region of the APOAI promoter was analyzed by re-sequencing in 549 Kuwaiti samples. DNA was extracted from blood taken from 549 healthy Kuwaiti volunteers who had fasted for the previous 12 h. Univariate and multivariate analysis was used to determine allele association with serum lipid levels.

**Results:**

The target sequence included a partial segment of the promoter region, 5’UTR and exon 1 located between nucleotides −141 to +294 upstream of the APOAI gene on chromosome 11. No novel single nucleotide polymorphisms (SNPs) were observed. The sequences obtained were deposited with the NCBI GenBank with accession number [GenBank: JX438706]. The allelic frequencies for the three SNPs were as follows: APOAI rs670G = 0.807; rs5069C = 0.964; rs1799837G = 0.997 and found to be in HWE. A significant association (p < 0.05) was observed for the APOAI rs670 polymorphism with increased serum LDL-C. Multivariate analysis showed that APOAI rs670 was an independent predictive factor when controlling for age, sex and BMI for both LDL-C (OR: 1.66, p = 0.014) and TC (OR: 1.77, p = 0.006) levels.

**Conclusion:**

This study is the first to report sequence analysis of the APOAI promoter in an Arab population. The unexpected positive association found between the APOAI rs670 polymorphism and increased levels of LDL-C and TC may be due to linkage disequilibrium with other polymorphisms in candidate and neighboring genes known to be associated with lipid metabolism and transport.

## Background

Plasma lipids have an essential role in several metabolic systems including the endocrine, nervous and circulatory systems [1] and their levels are influenced by lifestyle factors such as diet, physical activity and smoking, as well as genetic factors [2,3]. Variation in plasma lipid levels, commonly referred to as dyslipidemia, is characterized by increased total cholesterol (TC), triglycerides (TG), and low-density lipoprotein (LDL) as well as decreased high-density-lipoprotein (HDL). Dyslipiemia is commonly present among people whose diet is high in fat and low in fiber [4]. Twin and family studies have shown that plasma lipid levels are particularly influenced by genetic factors with a widely reported variation of heritability ranging from 40-60% [2]. Since lipid levels may be under genetic control [2,5], the identification and characterization of genetic variants associated with plasma concentrations may provide some insight into the genotype-phenotype relationship [3] and allow for lifestyle adjustments to improve health. Several reports, including the Genome Wide Association Studies, have attempted to elucidate the genetic and molecular mechanisms of dyslipidemia based on measurements of plasma lipids and have identified a limited number of candidate genes and pathways relevant to lipid metabolism and/or transport [3,6-11].

The APOAI gene encodes a major protein component of HDL that forms a structural part of the lipoprotein particles with a primary function in signaling and targeting lipoproteins in the reverse cholesterol transport from peripheral tissues to the liver for its excretion. It is also an important cofactor for lecithin cholesterolacyltransferase (LCAT) which catalyzes the formation of plasma cholesterol esters in the plasma [12] for their transfer from peripheral tissues. The gene encodes a 267 amino acid polypeptide, synthesized in the liver and intestine, where it undergoes intracellular cleavage followed by extracellular proteolysis yielding the mature 243 amino acid apoA-I polypeptide [13]. The APOAI gene is part of the APOAI/CIII/IV/V gene cluster located in chromosome 11q23-24 spanning over 20 Kb [14]. This gene cluster has been shown to have a relationship to dyslipidemia with different polymorphisms in each gene having varying degrees of association with different plasma lipids. There is much controversy regarding the association of specific reported single nucleotide polymorphisms (SNPs) at the APOAI locus with dyslipidemia. Several SNPs have been documented that are reported to be associated to HDL and cholesterol levels, including g.-75G > A (rs670), g. + 83C > T (rs5069) and g. + 84G > A (rs1799837). The rs670 variation has been reported to enhance promoter function [15] with the minor allele (A) being associated with abnormal variation in lipid levels in several populations [11,16-21]. In contrast, studies have reported this variant having decreased promoter activity [22], while others showed no effect on promoter activity [23]. In addition, several studies have reported different mutations in the coding region of the gene to be associated with reduced levels of HDL-C [8,13,24-27]. Another variation slightly further downstream is a C to a T transition at position rs5069 which occurs simultaneously or independently of a second transition from a G to an A at position rs1799837 [15,17,19,28,29]. The minor allele T for rs5069 is associated with increased HDL-C [15-17,19,29,30] and decreased TG levels [18] while the minor allele A for rs1799837 appears to be associated with HDL-C levels [15,27] as well as with heart disease [27].

The exact polymorphisms responsible for the apparent influence of this locus on HDL levels in particular are not fully elucidated [31] and very few studies have found an association with other lipid levels. It is well established that differences among populations in the relative frequency of ‘susceptibility genotypes’ or environmental exposure may contribute to the utility of a genotype for predicting a trait within a particular population [32,33]. These kinds of genetic association studies with regards to APOAI have not been investigated in the Kuwaiti population and there are only limited reports on other Arab populations [34,35]. Traditionally, the Kuwaiti/Arab diet is high in fat, raising the risk factors of cardiovascular diseases such as diabetes mellitus, dyslipidemia, obesity and hypertension. Studies on the lipid profiles of the Kuwaiti population [36] have reported a higher prevalence of hypercholesterolemia in women (36.6%) than in men (30.2%) with hypertriglyceridemia being higher in men (44.1%) than in women (33.8%) The Kuwaiti population also ranks high worldwide in prevalence of overweight (80.4%), obesity (47.5%), and metabolic syndrome (36.2%) [37]. It is hypothesized that the reported high frequencies of dyslipidemia and their association to metabolic syndrome are the outcome of an interaction between established risk factors and genetic polymorphisms. The objective of the present study was to assess the association of several genetic polymorphisms in the promoter region of the APOAI gene locus found by re-sequencing, with variation in lipid levels, among a cohort of the healthy Kuwaiti population.

## Methods

### Study population

This study was undertaken within the guidelines set by the Declaration of Helsinki and was reviewed and approved by the Local Ethical Committee at Kuwait University. The cohort in this study (n = 549) included 213 male and 336 female ethnic Kuwaiti nationals with ages ranging from 18 to 72 y with a mean of 29 y (±0.54 SEM). Whole blood was collected, with informed consent, from healthy volunteers undergoing a routine visit at a regional polyclinic or at one of the major hospitals in Kuwait. The randomized sample included 154 individuals with increased levels of serum TC (>5.17 mmol/L), 11 with increased TG levels (>2.19 mmol/L), 85 with increased levels of LDL-C (>3.2 mmol/L) and 113 with abnormal HDL-C levels (≤1.3 mmol/L). For each individual, several phenotypic variables including BMI (Table 1) and family history of hypercholesterolemia, hypertension, diabetes and heart disease were recorded.

**Table 1:** Demographic and clinical features of study cohort

**Parameters**	
Sex (Males, Females)	38.8%, 61.2%
Age (yr)	29 ± 0.54
BMI	27 ± 0.29
Cholesterol (mmol/L)	4.6 ± 0.03
LDL (mmol/L)	3.05 ± 0.03
HDL (mmol/L)	1.18 ± 0.01
Triglyceride (mmol/L)	0.92 ± 0.03
Positive family history of hypercholesterolemia	32.42%
Positive family history of heart disease	33.88%

Data shown as means ± SEM or for the BMI and lipid levels except for TG where the median and inter-quartile range is represented.

### Biochemical analysis and diagnostic criteria

A venous blood sample of 11 ml was obtained from all individuals who had previously fasted for at least 12 h. About 3 ml was used to determine serum lipid levels and 5 ml were transferred to tubes with anti-coagulant and used to extract genomic DNA. The levels of serum TC, TG, HDL-C, and LDL-C in each sample were determined by enzymatic methods with commercially available kits and performed on a UniCel DxC 800 Synchron Clinical System from Beckman Coulter (USA) in the Clinical Chemistry facility at Al-Amiri Hospital. Individuals were considered normal if their serum values for TC, TG, HDL-C and LDL-C fell within the Kuwaiti population reference range of 3.0–5.17, 0.40–2.19, 0.91–1.3 and 1.8–3.2 mmol/L, respectively. The cohort was divided into two groups normal and abnormal by each lipid parameter. Normal weight, overweight and obesity were defined as a BMI < 24, 24–28, and > 28 kg/m^2^, respectively.

### Re-sequencing of the partial region in the promoter/exon 1 of the APOAI gene locus

A 435 bp region of the APOAI promoter was analyzed by direct sequencing in 549 Kuwaiti samples. Genomic DNA was extracted from 5ml of whole blood using a proteinase K digestion and salting out extraction method as previously described by Miller et al. [38]. The purified DNA samples were first subjected to PCR amplification of the target sequence in the promoter region with the following primers: (forward) 5′-AGGGACAGAGCTGATCCTTGAACTCTT AAG −3′ and (reverse) 5′-TTAGGGGACACCTAC CCGTCAGGAAGAGCA-3′ using Fast Amplitaq Gold PCR Master Mix 1.25 ml in an Applied Biosystems Fast thermal cycler (Version 1.01, Life Technologies). An initial denaturation step of 95°C for 10 sec was followed by 35 cycles of denaturation at 94°C for 10 sec, 55°C for 25 sec, a final extension of 72°C for 30 sec and a subsequent hold at 4°C. The amplified PCR products (10 μl) were then purified with a Nucleospin column followed by formaldehyde denaturation. Sequencing was performed twice in two separate reactions, one with the forward primer and the other with the reverse primer for all samples in Fast thermal cycler (Life Technologies, Applied Biosystems) with an initial denaturation of 96°C for 1 min followed by 25 cycles of denaturation at 96°C for 10 sec, 50°C for 5 sec and 60°C for 1.15 min and a hold at 4°C.

The sequence extension products were then mixed with Xterminator solution (10ul) and Sam solution (45 ul) from the Big Dye Xterminator purification kit (Life Technologies, Applied Biosystems) and analyzed by capillary electrophoresis on the ABI Gene Analyzer 3130xl (Life Technologies, Applied Biosystems). The sequence data was documented on electropherogram files and subjected to analysis by the data collection software package (Version 3.0).

The sequences from the two reactions were compared for quality assurance and aligned by ClustalW (Multiple Alignment Tool) to screen for all novel and/or mutations. Multiple sequence alignment among all samples and in comparison to the reference sequence revealed only three APOAI polymorphisms (rs670, rs5069 and rs1799837) out of the 11 reported variants in the Genbank databases. (http://www.ncbi.nlm.nih.gov/gene).

The genotypes of the APOAI rs670, rs5069 and rs1799837 were also determined by sequence alignment with the published sequences [NCBI RefSeq: NT_033899.8] in the Genbank database for the promoter region of the APOAI employing BLAST sequence analysis (http://www.ncbi.nlm.nih.gov/gene).

### Statistical analysis of the association between the APOAI SNP’s and serum lipid levels

The allele and genotype frequencies were determined by simple gene-counting method for the polymorphisms including the APOAI rs670, rs5069 and rs1799837 and correlated with the individual phenotypic variables including serum lipid levels and documented family history of various complex diseases expressed as mean ± SEM, median, interquartile range and percentage where appropriate (Table 1). Hardy-Weinberg equilibrium (HWE) was tested using the GENEPOP [39] software (Version 4.0.10) with significance at p < 0.05. The relation between APOAI variants and lipid levels were evaluated by the Kruskal-Wallis ANOVA and the Mann–Whitney *U* tests where appropriate and multiple linear regression, using SPSS (version 19.0). A two-tailed p < 0.05 was considered as statistically significant. Multivariate analysis using logistic regression was used to identify the predictor factors expressed by odds ratio (OR) with 95% confidence intervals using R software environment (R Version 2.7.1) [40]. To estimate the power of the study, the sample size of 549 was analyzed using the Power and Sample Calculation Program (version 3.0.43) [41].

## Results

### Sequence analysis and molecular screening

**Figure 1:**
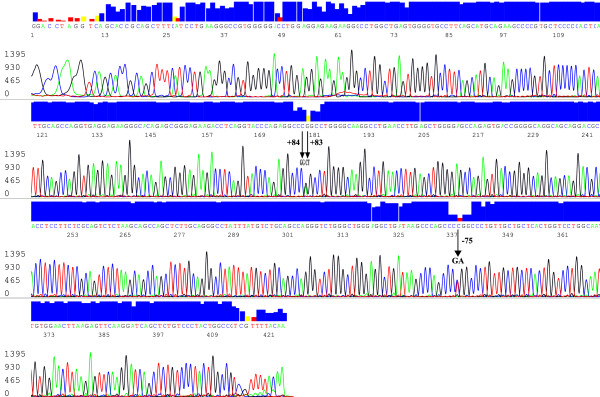
**An electropherogram of the 435 bp sequence data for the promoter region at the APOAI gene locus generated by the reverse primer.** The peaks represent the various nucleotides detected which are discriminated by the fluorescent color. The blue box on top is the quality assurance (95%) of the base call. The SNPs genotyped are indicated with and an arrow at positions −75, +83, +84 on the figure. The red and yellow box shown above the arrow had quality assurance <95% as a result of heterozygosity at those positions and was confirmed by sequence alignment with the sequence generated by the forward primer. The Kuwaiti individual in the figure was genotyped as a heterozygote GA for rs670, CT for rs5069 and homozygote GG for rs1799837. This sequence has been deposited along with the other 548 in the NCBI Genbank (http://www.ncbi.nlm.nih.gov/Genbank) with accession number [JX438706].

The 435 nucleotide sequence which included a partial segment of the APOAI promoter region, 5’UTR, intron and exon 1 located between nucleotides −141 to +294 upstream of the human APOAI gene at contig nucleotide position 20270896 – 20270462 of chromosome 11 (Figure 1) was analyzed for all 549 samples and aligned with the reference sequence to genotype the three SNP’s and to screen for putative polymorphisms. No novel SNP’s were observed; the sequences obtained were deposited in the NCBI Genbank with accession number [GenBank: JX438706]. The most common genotypes observed for the APOAI rs670, rs5.69, rs1799837 polymorphisms were those for the homozygote wildtype allele (Table 2). The least common genotype for the rs670 polymorphism was homozygote AA (3.8%) for the minor allele. Only one individual was observed with the homozygote TT for rs5069 while only three heterozygotes were observed for the rare allele rs1799837 and no homozygotes were identified. The allelic frequencies for all three SNP’s were as follows: APOAI rs670G = 0.807; rs5069C = 0.964; rs1799837G = 0.997. The genotype and allele frequencies at all three loci were found to be in HWE.

**Table 2:** Genotype and allele frequencies of the APOAI promoter region polymorphisms

**SNP**	**Genotype**	**n**	**%**	**Allele**	**Allele frequency**
APOA1 rs670bp	GG	358	65.20		
	GA	170	31.00	G	0.807
	AA	21	3.80	A	0.193
APOA1 rs5069	CC	511	93.10		
	CT	37	6.70	C	0.964
	TT	1	0.20	T	0.360
APOA1 rs1799837	GG	546	99.45		
	GA	3	0.55	G	0.997
	AA	0	0	A	0.003

### Association between the APOAI SNP’s and serum lipid levels

**Figure 2:**
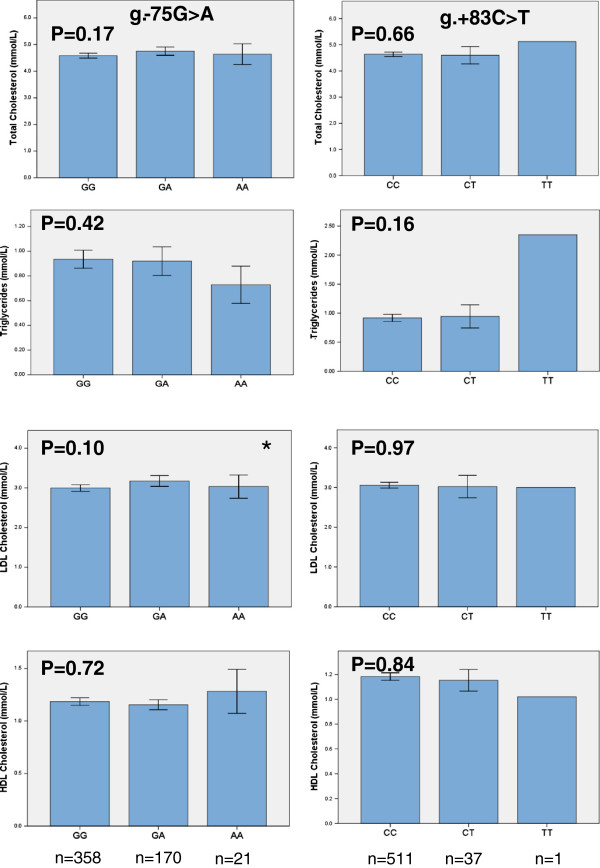
**Mean lipid levels as function of two APOAI SNPs, rs670 (g.-75G > A) and the rs5069 (g. + 83C > T).** Values shown are the mean ± SEM. p values were estimated according to Kruskal-wallis ANOVA and are shown. The Mann–Whitney U test was used to compare wild type genotypes against heterozygous and mutant genotypes combined, *, p < 0.05.

The relationship between genotype frequencies and serum levels of TC, TG, LDL-C and HDL-C are presented in Figure 2. A significant association (p = 0.02) was observed for the APOAI rs670 polymorphism with carriers of the A allele (n = 185) displaying higher LDL-C levels than individuals with the homozygote GG genotype (n = 344) (Table 3). An association (p = 0.03) was also found between the APOAI rs670 polymorphism and TC levels with carriers of the A allele (n = 191) displaying higher TC levels than individuals with homozygous GG genotype (n = 358) (Table 3). Neither HDL nor TG were significantly associated (p > 0.05). In a multivariate analysis using logistic regression the APOAI rs670 was found to be an independent predictive factor when controlling for age, sex and BMI for both TC and LDL-C levels with an odds ratio of 1.77 (95% CI: 1.17-2.69, p = 0.006) and 1.66 (95% CI: 1.10-2.51 p = 0.014), respectively (Table 4). No significant association was observed between the genotypes of the APOAI rs5069 and rs1799837 polymorphisms with regards to all the parameters analyzed including serum lipid levels and family history of dyslipidemia (data not shown).

**Table 3:** Association of the APOAI rs670 (g.-75G > A) and the rs5069 (g. + 83C > T) polymorphisms and lipid profiles

**SNP**		**W/W**		**W/M**		**M/M**	**p**
	n		n		n		
**SNPs relation with LDL**							
**rs670**	344	2.99 ± 0.04	164	3.17 ± 0.06	21	3.03 ± 0.14	**0.02**
**rs5069**	491	3.07 ± 0.03	37	3.02 ± 0.14	1	3.00	0.59
**SNPs relation with TC**							
**rs670**	358	4.58 ± 0.04	170	4.75 ± 0.07	21	4.64 ± 0.19	**0.03**
**rs5069**	511	4.64 ± 0.04	37	4.60 ± 0.16	1	5.12	0.78
**SNP relation with HDL**							
**rs670**	344	1.18 ± 0.01	164	1.15 ± 0.02	21	1.28 ± 0.1	0.22
**rs5069**	491	1.18 ± 0.01	37	1.15 ± 0.04	1	1.02	0.79
**SNP relation with TG**							
**rs670**	358	0.74	170	0.72	21	0.68	0.93
		(0.49–1.21)		(0.48–1.14)		(0.40–0.95)	
**rs5069**	511	0.72	37	0.78	1	2.35	0.83
		(0.48–1.18		(0.53–1.15)			

W/W, homozygous for common allele; W/M heterozygous for minor allele; M/M, homozygous for minor allele. The lipid level values are represented as mmol/L. Data shown as means ± SEM or for the lipid levels except for TG where the median and inter-quartile range is represented. p values were derived from multiple linear regressions with adjustment for sex, BMI and age assuming an additive genetic model. Significant values (p < 0.05) are shown in bold.

**Table 4:** Association of the APOAI rs670G > A genotype with total cholesterol and LDL-C

**Variable**	**OR 95% CI**	**P-value**
	**A. Cholesterol grouped (High vs Normal)**	
APOA rs670	1.77 (1.17–2.69)	0.006
Age	1.02 (1.00–1.03)	0.002
Sex	1.12 (0.74–1.70)	0.579
BMI	1.05 (1.02–1.09)	0.0004
	**B. LDL grouped (Normal vs Abnormal)**	
APOA rs670	1.66 (1.10–2.51)	0.014
Age	1.02 (1.00–1.03)	0.003
Sex	1.77 (1.18–2.65)	0.005
BMI	1.05 (1.02–1.08)	0.0006

The values represented include the odds ratio, confidence interval and p values as evaluated by multivariate analysis using logistic regression in 549 Kuwaiti individuals grouped based on their TC and LDL-C values according to the population normal reference values. Multivariate analysis shows the APOAI rs670 to be a significant (p < 0.05) independent predictive factor when controlling for age, sex and BMI for both cholesterol and LDL levels.

## Discussion

The present study is the first to report sequence analysis of the APOAI promoter region in an Arab ethnic group among the Kuwaiti population. Previous studies on the association of APOAI polymorphisms and variation in serum lipid levels have relied mainly on PCR-RFLP profiling [11,16,20,24,29,31,35] while a few studies have performed re-sequencing [13,19,42,43]. No novel sequences were observed in the sampled Kuwaiti population nor was the previously reported APOAI −27 polymorphism [13] in a Japanese boy, suggesting this one to be ethnic specific, not having been reported in many other populations. The other reported SNPs (CR991531, HR971651, rs12721302, rs372263101, rs199729971, rs141383703, rs368534370 and CS991294) in the target region sequenced were not identified in any of the Kuwaiti samples probably as a consequence of these SNPs being very rare (<0.01) in a given population and could be ethnic specific. In addition, the absence of novel SNPs in the Kuwaiti population, comprising mainly of Arab ethnicity, could reflect the biological importance of conserving the promoter region and the 5’ upstream sequence from the first transcription site at the APOAI gene locus. This would then ensure the integrity of its biological function in regulating APOAI gene expression, effecting regulation of gene expression of neighboring genes in the same cluster on chromosome 11, or influencing the expression of other apolipoprotein gene family. The integrity of the sequence upstream of the transcription site has also been reported to be essential to achieve sufficient expression of apoA-I [42,44] as various mutations in the promoter region result in marked reduction of APOAI expression and serum protein levels [44]. Moreover, sequence analysis of the promoter region has allowed identification of polymorphisms to be associated with extremely low concentrations of HDL-C. Matsunaga et al. [13] showed that compound heterozygosity of mutations at the promoter region (−27 and −75) resulted in significantly reduced levels of APOAI expression subsequently leading to reduced levels of apoA-I and HDL-C. Henakhaus et al. [43] also conducted sequence analysis of 94 South Asian immigrants in the USA and reported several polymorphisms in the APOAI gene (1683 bp), including the APOAI rs670. Specific hereditary patterns were common in this ethnic group, and that their frequencies were different from Europeans, suggesting the interaction of race and ethnicity on APOAI gene expression. Haase et al. [19] conducted a molecular analysis of the APOAI gene locus in over 10000 Danish individuals and reported a positive association between the APOAI −75 polymorphism and LDL and TC levels, and that homozygosity of APOAI rs670 A and heterozygosity of the APOAI rs5069 polymorphism were associated with increased HDL-C and apoA-I levels. This is consistent with our observation that the APOAI rs670 polymorphism is correlated with serum TC and LDL-C. In many of the studies reviewed, carriers of the minor A allele had significantly increased levels of LDL when compared with homozygote individuals with the wildtype G allele implicating it as a “risk” allele. On the other hand, our analysis of the APOAI rs670 and rs5069 genotypes with variation in HDL-C levels was in disagreement with Haase et al. [19] which may be due to the limitation in our sample size and ethnic variation. Several studies [13,19,24,31,32,42,44] have reported inconsistent results with regard to the effect of the APOAI polymorphisms including rs670 on HDL-C levels and its actual association with serum lipid levels. In comparison, Daneshpour et al. [29] documented an association between APOAI haplotypes consisting of the A allele with variation in serum TC and LDL-C levels. It has been suggested that the A allele is not directly responsible for the associative effect on serum APOAI and HDL-C levels [13,24] but rather is in play with other polymorphisms through linkage disequilibrium to regulate apoA-I levels [31] thereby affecting variation in serum HDL-C and TC levels. This may very well explain the results obtained in this study where carriers of the APOAI rs670 A allele were 1.77 times more at “risk” of increased TC levels (p = 0.006) and 1.66 times more likely to have increased LDL-C (p = 0.014) than those with the GG genotype when controlling for age, sex and BMI in the Kuwaiti population. The biological importance of APOAI rs670 is in its creation of an inverted sequence repeat homologous to other functional regulatory elements; such a mutation could have an important effect on the regulation by transcriptional factors [42] that may also affect other gene loci. Furthermore, the positive association (60.2% power calculation estimate with a 5% significance) observed between the APOAI rs670 and increased levels of LDL-C and TC, is more likely to be due to linkage disequilibrium with other polymorphisms in candidate and neighboring genes of lipid metabolism and/or transport such as APOB and LCAT which could be ethnic specific. This may somehow hinder the metabolism of LDL-C by the peripheral tissues leading to the failure of cholesterol esters entering the reverse cholesterol pathway and subsequently result in increased serum TC and LDL levels. However, no significant association was found with variation in lipid levels in the Kuwaiti population as indicated by the univariate analyses which is in consistent with others who did not observe an association between these changes that affect the MspI restriction site polymorphisms on HDL-C levels [32,45-49]. Wang et al. [16], however, reported a significant association of HDL-C with two SNPs (rs5069 and rs1799837) in which both were reported to be in linkage disequilibrium. Inconsistent results especially for the rs5069 and rs1799837 may sometimes arise from genotyping errors by PCR-RFLP due possibly to their adjacent positions as well as to several other factors. It has been suggested that the majority of the RFLPs are due to neutral mutations which probably do not directly affect the structure of the mature protein or the regulation of its neighboring genes [42]. This may also be the reason for the limited number of studies analyzing the +84 polymorphism. Nonetheless, this study further supports the designation of the APOAI rs1799837A allele as a “rare” allele.

The Kuwaiti population is heterogeneous comprised of a major Arab ethnic group including mostly bedouins, a minor Iranian ethnic group and admixes of other ethnicities [50]. Kuwait is a country that was first inhabited during the early 18^th^ century by Arab nomads and merchants migrating from the Arabian Peninsula, Mesopotamia and East Asia [50]. The origins of most of the current population of more than one million can be traced to three distinct regions now known as Saudi Arabia, Iran and Iraq comprising mainly Arabs whose ancestors were from the Arabian Peninsula and directly settled in Kuwait in the 19th century [50]. More recently, migrants from different parts of Asia (e.g. Jordan, Lebanon, and Syria, India) and North East Africa (e.g. Egypt and Greater Morocco) have also settled in Kuwait leading to increased admixture between the different subpopulations. Based on the analysis of the APOAI promoter region, the genetic contribution from each ethnic group into the Kuwaiti general gene pool is minimal that it doesn’t appear to have made much difference in the distribution of the genotypes among the different subpopulations (p > 0.05). Moreover, variation in lipid levels as a result of the contribution of other Arab ethnic groups in the Kuwaiti population is also minimal as the number of samples with such ethnicity is low (< 5%) therefore does not appear to have affected the normal distribution of TC, HDL and LDL levels. This makes it difficult to conduct analysis on the genotypes of such ethnic groups and their association to variation in HDL levels.

The genotype analysis of the three polymorphisms in the studied Kuwaiti population and in the two major ethnic groups showed the genotype and allele frequencies to be in HWE suggesting any positive finding to be more likely related to the biological function of the APOAI rs670 than to population stratification. In addition, the contribution made by the Kuwaiti-Iranian subpopulation (22.77%) did not result in any significant difference to the overall population genotype frequency distribution. The allele frequency distribution at the three loci was compared to other reported populations (Table 5). The allelic frequency in the Kuwaiti population was found to be 19.3% at the APOAI rs670 which falls between the low 10% frequency reported for Africans [17] and for Iranians (13.8%) [26] and the high 63.6% in Chinese [11] while being approximately similar to those of Omani Arabs, Caucasian and Europeans (15-22%) [15,24,35] as well as being very close to the Danish [19]. However, the observed +83T frequency (3.6%) is lower to the range of 5.1-7.8% reported for Omanis and Iranians (18,27) and much lower than that of the African population (17) yet similar to the 3.5-4.0% reported in the Europeans Danes (19). The reported frequencies suggest demographic and gene flow patterns which need further investigation. Li et al. [20] also reported that variation in allelic frequencies at the APOAI rs670 between two subpopulations in China and the rare allelic frequency was different from Caucasians while being lower than in other oriental races suggesting the utility of this SNP in distinguishing various races. Those similar allelic frequencies could be attributed to a common ancestor homology. The inter-population sequence variation and allelic frequencies in the APOAI promoter region could variably modulate APOAI expression [16,19,22,23,32] subsequently leading to effects on serum lipid levels that manifest themselves in different ways in different populations. A GWA study conducted by the Global Lipids Genetic Consortium [51] in Europeans did not report any association of the three analyzed SNPs in the present study with variation in lipid levels as they were not covered by any of the microarray platforms used in the analysis. However, the study reported an association with SNP rs964184 [51]. To investigate if the two studied SNPs (rs670 and rs5069) were in LD with the published SNP, the Proxy SNPs rs11216153 and rs7123326 used in the Hapmap and 1000genome data was used to tag the two SNPs. The analysis showed that rs964184 was not in LD with rs670 and rs5069 (r^2^ = 0.116 and r^2^ = 0.006 respectively). Likewise, the rs9326246 recently published by the CardioGramplusC4D [52] cconsortium was tagged by another Proxy SNP rs7350481 and was found not to be in LD with rs11216153 and rs7123326 (r^2^ = 0.015 and r^2^ = 0.005 respectively).

**Table 5:** Comparison of the APOAI promoter rs670 and rs65069 allelic frequencies reported in this study with other populations

**Population**	**Sample size**	**SNP allele frequency**				**Reference**
		**rs670**		**rs5069**		
	**n**	**G**	**A**	**C**	**T**	
Africans (Nigeria)	786	0.899	0.101	0.598	0.402	Kamboh et al. [17]
Caucasians	243	0.779	0.221	0.959	0.041	Wang et al. [16]
Europeans	1078	0.841	0.159			Talmud et al. [24]
Europeans (Danish)	190*	0.810	0.190	0.965	.035	Haase et al. [19]
	10273	0.810	0.019	0.960	.040	
French Canadians	653	0.850	0.150			Minnich et al. [31]
Brazilians	414		0.180			De Franca et al. [32]
Chinese	1030	0.364	0.636	-	-	Yin et al. [11]
Hei Yi Zhaung Chinese	474	0.703	0.298	-	-	Li et al. [20]
Han Chinese	564	0.660	0.340	-	-	Li et al. [20]
Hong Kong Chinese	271	0.689	0.311	0.949	0.051	Ma et al. [18]
Japanese	1880	0.844	0.156	0.922	0.078	Shioji et al. [27]
Iranian	823	0.862	0.138	0.946	0.054	Daneshpour et al. [29]
Omanis	150	0.783	0.217	0.933	0.067	Al-Yahyaee et al. [35]
Kuwaitis	549	0.807	0.193	0.964	0.036	Present study

*Based on re-sequencing of the APOAI promoter region [19].

## Conclusion

In this study, three specific polymorphisms were genotyped by re-sequencing the APOAI promoter region including the common rs670 polymorphism along with the rare polymorphisms at the +83 and +84 loci in a single reaction, overcoming problems with genotyping errors frequently observed with PCR-RFLP. The present study showed that the Kuwaiti population could be genetically disposed to developing dyslipidemia in the presence of the APOAI rs670 polymorphism and other SNPs which need to be further investigated. Re-sequencing was found to be a more informative option, especially if inter-population variation and ethnicity have a role in the genetic association of the gene and its outcomes. With recent advances and the availability of high performance, cost effective automated gene analyzers such as Next-Generation sequencing, conducting high throughput genotyping and mutation screening of candidate genes in lipid metabolism and/or transport is highly recommended for different ethnic groups and in a larger sample size in order to better understand the differences in population frequencies, and to more accurately define “risk” and “protective” alleles from those which are simple variants. This will assist in the goal towards personalized and preventive medicine.

## Abbreviations

apoA-I: Apolipoproteinai protein; APOAI: Apolipoprotienai gene; APOB: Apolipoproteinb gene; BLAST: Basic local alignment search tool; BMI: Body mass index; DNA: Deoxyribonucleic acid; HDL-C: High-density-lipoprotein-cholesterol; LCAT: Lecithin cholesterolacyltransferase; LDL-C: Low-density lipoprotein-cholesterol; NCBI: National center for biotechnology information; OR: Odds ratio as estimated by logistic regression; PCR: Polymerase chain reaction; SNPs: Single nucleotide polymorphisms; TC: Total cholesterol; TG: Triglycerides.

## Competing interests

The authors declare there are no competing interests.

## Authors’ contributions

SA prepared the project proposal and study design, supervised the molecular genetic studies and sample collection and documentation, participated in the sequence alignment and analysis and drafted the manuscript. AA participated in the study design, carried out most of the statistical analysis and assisted in draft of the manuscript. BA carried out the molecular genetic techniques, sequencing and sequence alignment and participated in the manuscript draft. MA contributed to the study design, participated in the data analysis and edited the manuscript. GE facilitated sample collection and documentation of the clinical and phenotypic data and assisted with the lipid profile analysis. All the authors have read and approved the final manuscript.

## Pre-publication history

The pre-publication history for this paper can be accessed here:

http://www.biomedcentral.com/1471-2350/14/90/prepub
